# A CNL protein forms an NLR pair with NRCX to modulate plant immunity

**DOI:** 10.1007/s44154-025-00245-6

**Published:** 2025-09-03

**Authors:** Xiaohua Dong, Xiaoyan Zhang, Xu Lu, Yufeng Yang, Chuyan Xia, Weiye Pan, Zhiyuan Yin, Yaning Zhao, Gan Ai, Daolong Dou

**Affiliations:** College of Plant Protection, Academy for Advanced Interdisciplinary Studies, Nanjing Agricultural University, Nanjing, China

**Keywords:** NRCX/NARY, Paired NLR, Growth-defense trade-off

## Abstract

Nucleotide-binding leucine-rich repeat (NLR) proteins assemble into genetically linked pairs to mediate effector-triggered immunity (ETI) in plants. Here, we characterize the paired NLRs *NRCX* and *NARY* (NRCX adjacent resistance gene Y) in *Nicotiana benthamiana*. CRISPR/Cas9 knockout of *NRCX* caused severe dwarfism and constitutively activated immunity, marked by *PR1* upregulation and enhanced resistance to *Phytophthora capsici*. Co-silencing or double knockout of the adjacent NLR *NARY* partially rescued the *nrcx* phenotype, revealing NARY as a compensatory regulator that modulates growth and immunity. Structural analysis revealed that NARY harbors non-canonical Walker B and MHD motifs, which lack autoactivation capacity despite their divergence from canonical NLR executors. Split-luciferase and co-immunoprecipitation assays showed that NRCX and NARY interact exclusively through their CC domains, forming a non-canonical regulatory complex. Notably, simultaneous silencing of *NRC2/3* and *NARY* incompletely restored growth in *nrcx* mutants, implicating additional factors in immune modulation. Our findings establish NARY as a compensatory NLR partner of NRCX that fine-tunes immunity without triggering cell death, revealing a novel mechanism for balancing growth and defense in Solanaceae.

**Supplementary Information:**

The online version contains supplementary material available at 10.1007/s44154-025-00245-6.

## Introduction

Plants have evolved a multi-layered immune system to defend against microbial pathogens, with intracellular nucleotide-binding leucine-rich repeat (NLR) proteins serving as critical sensors for pathogen detection. These receptors recognize pathogen-derived effectors through direct or indirect interactions, initiating effector-triggered immunity (ETI), a robust defense response often accompanied by localized programmed cell death, a process known as the hypersensitive response (HR), to limit pathogen spread (Dodds and Rathjen 2010; Jones and Dangl 2006). A key feature of NLR-mediated immunity is the formation of genetically linked pairs, where a sensor NLR detects effectors and associates physically with a partner executor NLR to transmit immune signals (Cesari et al. 2014). Executor NLRs often contain degenerated nucleotide-binding motifs, such as Walker B and MHD, making them dependent on their cognate sensors to avoid autoactivation while enabling rapid signal amplification upon effector recognition (Marchal et al. 2022; van Ooijen et al. 2008).

The Solanaceae family displays a unique evolutionary pattern in NLR biology, characterized by the expansion of NRC (NLR required for cell death) helper networks (Goh et al. 2024; Sakai et al. 2024). These NRC proteins act as central signaling nodes, integrating inputs from diverse sensor NLRs to coordinate defense responses (Wu et al. 2017; Zhu et al. 2019). Yet, how genetically paired NLRs have become functionally specialized within this lineage remains unclear. In non-Solanaceae species, canonical executor-sensor pairs like rice RGA4/RGA5 depend on conserved motifs and hierarchical interactions (Cesari et al. 2014). In contrast, Solanaceae NLR pairs may have evolved distinct regulatory strategies, potentially diverging from these conserved mechanisms to interact with the lineage-specific NRC networks (Adachi et al. 2019; Wu et al. 2017). Previous research identified that knockout of *NRCX*, an atypical NLR modulator in *Nicotiana benthamiana*, leads to severe dwarfism, suggesting its role in balancing growth and immune responses (Adachi et al. 2023). Unlike canonical NRC helper NLRs such as NRC2/3/4, NRCX functions as a modulator rather than a core signaling hub, but the molecular pathways through which it influences plant growth remain unclear.

NLRs share a basic protein architecture, consisting of a variable N-terminal domain, a central nucleotide-binding domain (NB-ARC), and a C-terminal leucine-rich repeat domain (LRR) (Takken and Goverse 2012). The NB-ARC domain can be further subdivided into three subdomains: NB, ARC1, and ARC2 (Takken and Goverse 2012). The NB subdomain contains two major motifs: a P-loop motif required for nucleotide binding and a Walker B motif required for adenosine triphosphate (ATP) hydrolysis (Kim et al. 2005). Additionally, the ARC2 subdomain harbors an MHD motif (methionine-histidine-aspartate) within the nucleotide-binding site (Williams et al. 2011). Mutations in the Walker B or MHD motifs of NLRs frequently result in constitutive autoactivation (van Ooijen et al. 2008). For instance, substituting the conserved second aspartate with glutamate in I-2 reduces ATP hydrolysis rates and triggers autoactivation. Notably, many executor NLRs exhibit non-canonical MHD motifs, which are associated with their autoactivation capacity (van Ooijen et al. 2008). For example, the MHD motif of RGA4 is "TYG", a highly divergent variant that differs from the canonical MHD sequence. Replacing this divergent motif with a canonical MHD motif abolishes RGA4 autoactivity (Cesari et al. 2014).

Here, we investigate a previously uncharacterized NLR pair in *N. benthamiana*. NRCX and its adjacent head-to-head partner, NARY. Building on prior work showing that *NRCX* loss causes dwarfism (Adachi et al. 2023), our study demonstrates that co-silencing *NARY* partially rescues the developmental and immune phenotypes of *nrcx* mutants, indicating a compensatory regulatory partnership distinct from classical executor-sensor pairs. Structural analyses reveal that NARY harbors non-canonical Walker B and MHD motifs yet lacks the ability to induce autoactive cell death. Instead, NARY interacts with NRCX exclusively through their coiled-coil (CC) domains, forming a heterocomplex that modulates immunity without triggering HR. Our findings uncover a non-canonical regulatory mechanism in Solanaceae, where a novel regulatory mechanism in Solanaceae NLR biology, where atypical motif configurations and domain-specific interactions enable NLR pairs to balance immune activation with growth homeostasis, expanding the functional repertoire of plant immune receptors.

## Results

### NRCX knockout in *N. benthamiana* confers dwarfism and enhanced disease resistance

**Fig. 1:**
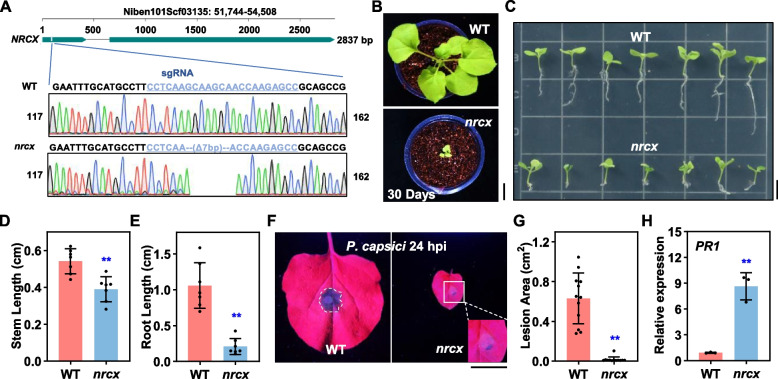
Growth defects and enhanced resistance in *nrcx* mutants. **A** Schematic of *NRCX* deletion in *Nicotiana benthamiana*. Genomic organization of *NRCX* (top) with the guide RNA (gRNA) target site and sequencing-confirmed 7-bp deletion in *nrcx* mutants (bottom). **B** Phenotypic comparison of wild-type (WT) and *nrcx* knockout plants after 30 days of soil cultivation. Bar = 2 cm. **C** Growth phenotypes of WT and *nrcx* seedlings on sterile 1/2 MS medium at three weeks post-germination. Bar = 2 cm. **D,E** Quantification of stem and root lengths in WT and *nrcx* mutants grown on 1/2 MS medium (means ± SD; *n* = 7; **, *P* ≤ 0.01; Student’s* t-*test). **F,G** Enhanced pathogen resistance in *nrcx* mutants. UV fluorescence imaging of *Phytophthora capsici*-inoculated leaves at 24 h post-inoculation (hpi) and lesion area quantification (means ± SD; *n* = 12; **, *P* ≤ 0.01; Student’s* t-*test). Bar = 2 cm. **H** *PR1* expression upregulation in *nrcx* mutants (means ± SD; *n* = 3; **, *P* ≤ 0.01; Student’s* t-*test)

During a genome-wide NLR silencing screen using virus-induced gene silencing (VIGS) in *Nicotiana benthamiana* (Dong et al. 2025), we observed pronounced dwarfism in plants silencing a subset of NLR genes, including NRCX (NLR-required for cell death X) (Fig. S1A,B, NbD037153.1 used as a control). While *NRCX*-dependent dwarfism was previously reported (Adachi et al. 2023), we extended these findings by generating *nrcx* knockout mutants via CRISPR/Cas9. These mutants confirmed that the phenotype was intrinsic to *NRCX* loss-of-function and not an artifact of VIGS or viral infection (Fig. 1A,B and Fig. S1C,D).

Strikingly, *nrcx* mutants maintained their dwarf stature even under axenic growth conditions (Fig. 1C), demonstrating that the phenotype arises spontaneously and is independent of microbiome interactions. This observation is consistent with the NLR deficiency-induced autoimmune phenotype reported in previous studies (Cheng et al. 2024). Tissue-specific analysis revealed stronger growth suppression in roots versus shoots (Fig. 1D,E), aligning with prior reports of elevated *NRCX* expression in root tissues (Adachi et al. 2023).

To probe the immunological consequences of *NRCX* disruption, we challenged mutants with the oomycete pathogen *Phytophthora capsici* (strain LT263). *nrcx* plants exhibited significantly enhanced resistance compared to wild-type controls (Fig. 1F,G). This heightened defense correlated with constitutive activation of immune markers, as evidenced by ~ ten fold upregulation of PR1 (Pathogenesis-Related Protein 1) transcripts in uninfected *nrcx* mutants (Fig. 1H).

### Simultaneous silencing of *NARY* partially rescues the *NRCX*-silenced plant phenotype

Knockout of one component in paired NLRs may activate their cognate NLR partner, triggering defense pathway induction and growth inhibition (van Wersch et al. 2020). We further characterized NRC helper NLRs and quantified paired NLRs across representative plant species (Fig. S2A,B). Similar to previous report, our data supported that NRC helper NLRs underwent lineage-specific expansion within the Lamiids clade of Asterids (Fig. S2A) (Goh et al. 2024; Kolli 2024; Sakai et al. 2024). *N. benthamiana* possesses only three NLR pairs: *NRCX* and its adjacent head-to-head partner, NARY (NRCX-adjacent resistance protein Y), *NbD002668.1/NbD002669.1*, and *NbD026701.1/NbD026704.1* (Fig. S2, S3 A). As paired NLRs typically function as units, knockout/knockdown of one partner may active the other, resulting in dwarfism and enhanced resistance phenotypes (Wang et al. 2019). Intriguingly, distinct dwarfism was observed exclusively upon *NRCX* silencing (Fig. S3B,C).

*NARY* and *NRCX* are arranged in a head-to-head orientation separated by an 18,795 bp intergenic region (Fig. S4A). We hypothesized that *NRCX* silencing might activate *NARY*, thereby inducing defense responses contributing to dwarfism. To test this, we co-silenced *NRCX* and *NARY* using a single TRV vector containing both genes (Fig. S4B). Compared to TRV-*NRCX* (TRV-*X*) plants, TRV-*NRCX/NARY* (TRV-*X/Y*) plants exhibited significantly reduced growth inhibition, though remained smaller than TRV-*GUS* (*β-glucuronidase*) controls (Fig. S4C-E).

Subsequent immune response evaluation revealed that *PR1* expression was markedly lower in *NRCX/NARY*-silenced plants compared to TRV-*X* plants (Fig. S4F). These findings suggest *NARY* positively regulates immunity and partially mediates dwarfism in *NRCX*-silenced plants.

### Double knockout of *NRCX* and *NARY* partially rescues *nrcx* phenotype

**Fig. 2:**
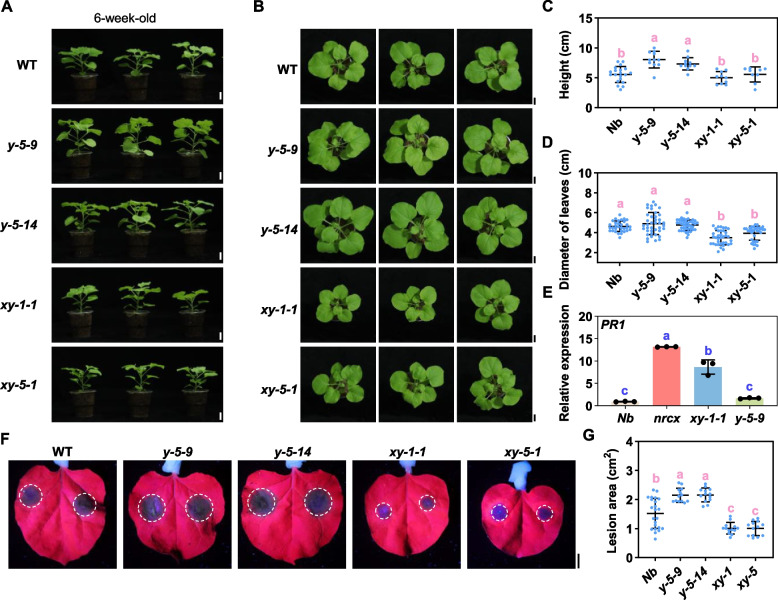
Knockout of *NARY* partially rescues *NRCX*-knockout phenotype. **A,B** Morphology of the indicated mutants. Photographs were taken 6 weeks after transplanting the plants. Bars = 2 cm. **C** Height of the indicated mutants. Data are means ± SD (*n* ≥ 9; lowercase letters indicate significant differences between groups as determined by one-way ANOVA, *P* < 0.05). **D** Leaf diameter of the indicated mutants. Data are means ± SD (*n* ≥ 32; lowercase letters indicate significant differences between groups as determined by one-way ANOVA, *P* < 0.05). **E** Detection of *PR1 gene* expression in indicated mutants (means ± SD; *n* = 3; **, *P* ≤ 0.01; Student’s *t-*test). **F** Photograph of mutant leaves inoculated with *P. capsici* zoospores at 36 hpi under UV light. Bars = 1 cm. **G** Lesion areas on the indicated leaves caused by *P. capsici*. Data are means ± SD (*n* ≥ 13; lowercase letters indicate significant differences between groups as determined by one-way ANOVA, *P* < 0.05)

Using CRISPR/Cas9, we generated double *NRCX/NARY* knockouts (xy-1–1 and xy-5–1) and single *NARY* knockouts (*y-5–9* and *y-5–14*) (Fig. S5A,B). T2 generation plants with stable inheritance were used for subsequent analyses. Double knockouts partially rescued the *nrcx* phenotype (Fig. 2A-D vs Fig. 1B), showing WT-compared height but significantly smaller leaves (Fig. 2A-D vs Fig. 1B). Notably, single *NARY* knockouts were taller than WT plants while maintaining the same leaf size (Fig. 2A-D), suggesting *NARY* may act as a negative growth regulator.

The relative expression levels of the *PR1* gene demonstrated significant upregulation in both the *NRCX* single knockout and *NRCX/NARY* double knockout mutants compared to the wild type (Fig. 2E). In contrast, the *NARY* single knockout mutant showed no significant difference in *PR1* gene expression relative to the wild type (Fig. 2E). Pathogen resistance assays using *P. capsici* inoculation revealed enhanced susceptibility in single *NARY* knockouts, displaying larger lesion areas than WT (Fig. 2F,G). Conversely, double knockouts exhibited improved resistance with reduced disease progression compared to WT (Fig. 2F,G), indicating *NARY* positively regulates immunity against *P. capsici*.

### Simultaneous silencing of *NRC2*, *NRC3*, and *NARY *fails to fully restore the phenotype of *NRCX*-silenced plants

Previous studies demonstrated that knockout of *NRC2* and *NRC3* partially rescues the dwarf phenotype caused by *NRCX* silencing (Adachi et al. 2023). To determine whether combined silencing of *NRC2*, *NRC3*, and *NARY* could fully restore the *NRCX*-silenced phenotype, we performed VIGS in wild-type *N. benthamiana* using TRV constructs targeting *NRC2/NRC3* (TRV-*2/3*), *NRC2/NRC3/NRCX* (TRV-*2/3/X*), and *NRC2/NRC3/NRCX/NARY* (TRV-*2/3/X/Y*) (Fig. S6A). Plants subjected to TRV-*2/3/X/Y* showed rescue of the dwarf phenotype compared to TRV-*2/3/X* plants, but failed to fully restore growth to the level observed in TRV-*2/3* controls (Fig. S6A-D).

To further validate these findings, we conducted parallel VIGS experiments in both wild-type and *nrc2/3/4* knockout mutants. Silencing *NRCX* alone (TRV-*X*) in wild-type and *nrc2/3/4* plants resulted in severe dwarfism, while co-silencing *NRCX* and *NARY* (TRV-*X/Y*) significantly improved growth in both genetic backgrounds (Fig. S6E). Notably, TRV-*X/Y*-treated wild-type and *nrc2/3/4* mutant plants exhibited comparable sizes, indicating that *NRC2/3/4* loss does not further enhance the phenotypic rescue (Fig. S6E). These results demonstrate that simultaneous silencing of *NRC2*, *NRC3*, and *NARY* incompletely rescues the *NRCX*-silenced phenotype, suggesting the involvement of additional regulators beyond these NLRs in modulating growth outcomes.

### *NRCX/NARY* pair is specific to Solanaceae species

**Fig. 3:**
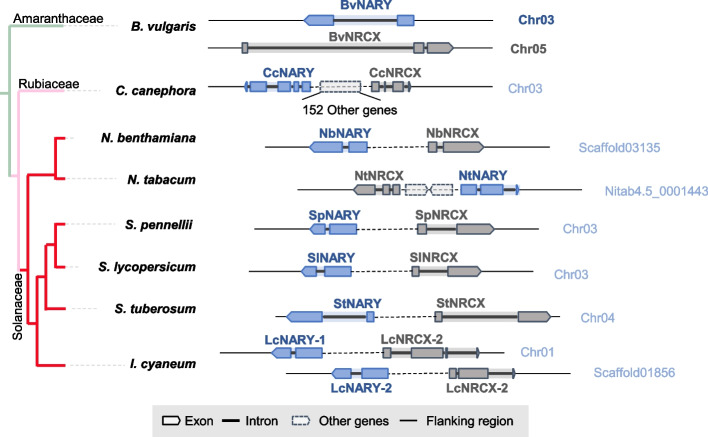
Schematic diagram of *NRCX* and *NARY* in plant genomes*.* Genomic organization of *NRCX* and *NARY* across different plant species, with phylogenetic relationships displayed as an evolutionary tree on the left. The relative positions and orientations of *NRCX* (gray) and *NARY* (blue) are shown

NRCX homologs in *B. vulgaris* occupy a phylogenetically basal position in the Asterid lineage (Fig. S7), consistent with the reported Asterid-specific distribution of NRCX (Adachi et al. 2023). *NARY* homologs showed broader conservation across dicots (Fig. S7). The *NRCX/NARY* locus exhibits distinct genomic organization across plant species. In *N. benthamiana*, *N. tabacum*, *Solanum pennellii*, *S. lycopersicum*, *S. tuberosum*, and *Ipomoea cyaneum*, NRCX and NARY are adjacent on the same chromosome (Fig. 3). In contrast, their homologs in *Beta vulgaris* reside on separate chromosomes, while those in *Coffea canephora* are spaced 152 genes apart. This conserved physical linkage of NRCX/NARY in Solanaceae species correlates with their compensatory functional interaction, as demonstrated by phenotypic rescue assays.

### The Walker B and MHD motifs of NARY are non-canonical

**Fig. 4:**
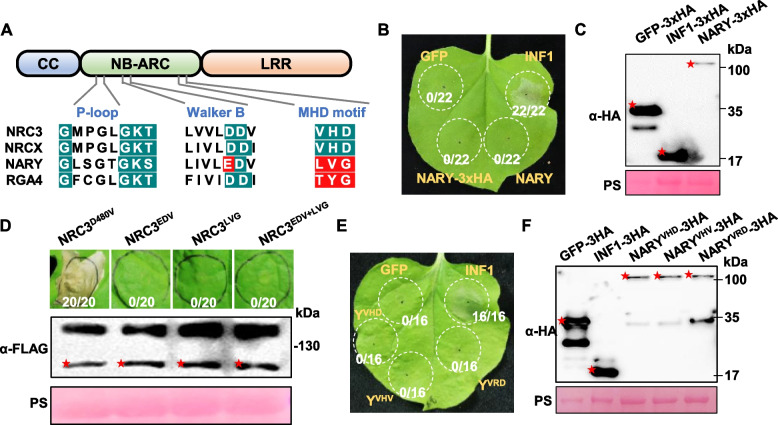
NARY does not induce hypersensitive response (HR) in *N. benthamiana*.** A** Sequence alignment of P-loop, Walker B and MHD motifs in NLR proteins (NRC3, NRCX, NARY, RGA4). Canonical residues are highlighted in blue, non-canonical residues in red. **B** Cell death phenotypes in leaves expressing NARY. GFP (negative control) and INF1 (positive control) were expressed for comparison. **C** Immunoblot analysis of NARY-HA protein accumulation in *N. benthamiana* leaves at 36 h post-agroinfiltration (hpi). Total protein loading was verified by Ponceau S staining. **D** Cell death induced by NRC3 mutants. Autoactive NRC3^D480 V^ (positive control) and chimeric mutants (NRC3^EDV^, NRC3^LVG^, NRC3^EDV+LVG^) carrying NARY motifs were analyzed. Protein levels of variants are shown below. **E** Phenotypes of leaves expressing NARY mutants (NARY^VHV^, NARY^VRD^) at 5 days post-infiltration (dpi). **F** Immunoblot confirmation of NARY mutant protein accumulation

The NB-ARC domain of NLRs contains critical motifs (P-loop, Walker B, MHD) required for ATP hydrolysis and autoactivation (van Ooijen et al. 2008). Sequence analysis of the 881-aa *NARY* CNL protein revealed a canonical P-loop (GxxxxGK[T/S]) but non-canonical Walker B and MHD motifs (Fig. 4A). The Walker B motif possesses a glutamate substitution at the conserved aspartate position, a mutation known to impair ATP hydrolysis (Tameling et al. 2006). The MHD motif is fully degenerate, with all three residues deviating from the consensus (Fig. 4A). Phylogenetic comparisons showed that *NARY* homologs in Arabidopsis, kiwifruit, and *B. vulgaris* retain canonical motifs, whereas partial degeneration occurs in *Coffea* homologs and complete degeneration in Solanaceae species (Fig. S7). These results suggest progressive degeneration of Walker B and MHD motifs during NLR evolution in Solanaceae, potentially uncoupling ATPase activity from autoactivation.

### NARY lacks autoactivation capacity despite degenerate motifs

To test whether NARY’s non-canonical motifs confer autoactivation, we transiently expressed the protein in *N. benthamiana*. No cell death was observed when expressing NARY in leaves, regardless of whether the protein was fused to a tag (Fig. 4B). Western blot analysis indicated that *NARY* was expressed correctly (Fig. 4C). These results suggest that, like NRCX (Adachi et al. 2023), NARY does not possess the ability to auto-induce cell death.

These results motivated us to investigate whether the Walker B and MHD motifs of NARY are auto-activating mutants. Domain-swapping experiments replacing Walker B and/or MHD motifs of NRC3 with those of NARY (generating NRC3^EDV^, NRC3^LVG^, and NRC3^EDV+LVG^) also failed to induce cell death, unlike the autoactive NRC3^D480 V^ control (Fig. 4D). Artificial restoration of canonical MHD motifs in NARY (NARY^VHV^, NARY^VRD^) similarly did not trigger cell death (Fig. 4E, F). These data demonstrate that NARY's degenerate motifs are not autoactivating mutations, implicating additional regulatory mechanisms for its activation.

### Physical interaction between NRCX and NARY via CC domains

**Fig. 5:**
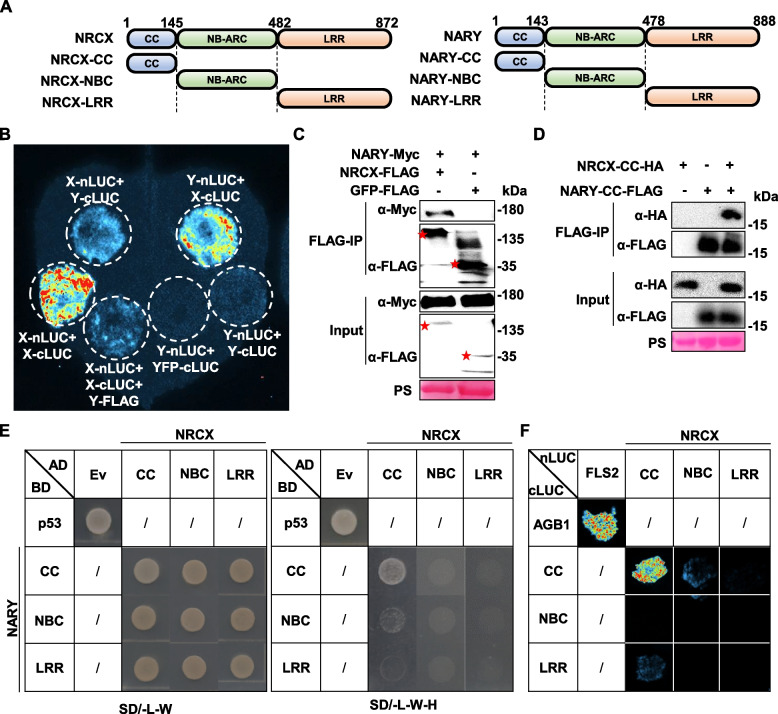
Interactions between NRCX and NARY.** A** Domain architecture of NRCX and NARY, highlighting coiled-coil (CC), NB-ARC (nucleotide-binding adaptor shared by APAF-1, R proteins, and CED-4), and leucine-rich repeat (LRR) domains. Domain boundaries (amino acid positions) are indicated in the schematic. **B** Split-luciferase complementation assay confirming NRCX-NARY interaction. Agrobacterium strains carrying NRCX-nLUC and NARY-cLUC were co-infiltrated into distinct leaf sectors (constructs labeled adjacent to infiltration sites). **C** Co-immunoprecipitation (Co-IP) demonstrates NRCX interacts with NARY. Total protein extracts from *N. benthamiana* leaves co-expressing NRCX-FLAG and NARY-Myc were immunoprecipitated with anti-FLAG beads, and probed with anti-Myc antibodies. **D** Co-IP reveals CC domain-mediated interaction. NRCX-CC-HA and NARY-CC-FLAG were co-expressed, immunoprecipitated with anti-FLAG beads, and detected via anti-HA antibodies. **E** Yeast two-hybrid (Y2H) assay validates NRCX-CC/NARY-CC interaction. The Ev/p53 pair served as a positive interaction control. **F** Split-luciferase assay further confirms NRCX-CC/NARY-CC interaction. FLS2-nLUC + AGB1-cLUC was included as a positive control

While paired NLR genes generally show genomic colocalization and physical interactions (Adachi et al. 2019; Jubic et al. 2019), the *NRCX/NARY* pair lacks hallmarks of canonical autoactivating NLRs. To explore functional crosstalk, we first interrogated their physical association using split-luciferase complementation assays. A strong interaction signal was detected between NRCX and NARY in planta (Fig. 5B). Intriguingly, NRCX exhibited self-complementation signal, whereas no self-complementation was detected for NARY (Fig. 5B). “NARY-nLUC + YFP-cLUC” as a negative control (Fig. 5B). Coexpression of NARY significantly attenuated the self-complementation signal of NRCX (Fig. 5B), suggesting competitive binding at the interaction interface. This heteromeric interaction was corroborated by co-immunoprecipitation (Co-IP) assays (Fig. 5C).

To map interacting domains, we systematically analyzed the CC, NB-ARC, and LRR domains through Co-IP, split-luciferase, and yeast two-hybrid (Y2H) assays (Fig. 5A). All approaches convergently demonstrated that the CC domains of NRCX and NARY exclusively mediated their interaction (Fig. 5D-F).

## Discussion

**Fig. 6:**
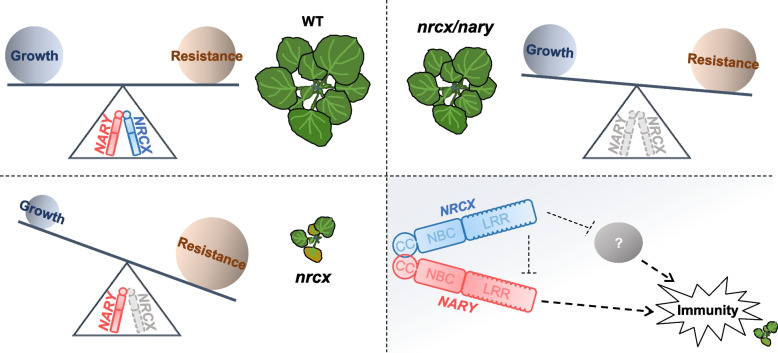
Schematic model of NRCX/NARY-mediated regulation of growth and immunity in *N. benthamiana*. In the depicted balance model, the fulcrum is jointly supported by *NRCX* (blue) and *NARY* (red), with knocked-out genes represented by gray models and text. The dark blue circle on the left side of the balance symbolizes “Growth,” while the dark yellow circle on the right represents “Resistance” of *N. benthamiana* against *P. capsici*. In wild-type *N. benthamiana*, Growth and Resistance remain balanced, as indicated by the identical diameters of the dark blue and dark yellow circles. The circle diameter reflects relative weight, with larger diameters corresponding to heavier weights that tilt the balance toward the respective side. Both NRCX and NARY contain CC, NBC, and LRR domains, with their CC domains interacting physically, illustrated by direct contact between their CC termini. Dashed lines denote hypothesized pathways: arrowheads indicate activation, while bar-ended lines represent suppression. A white question mark enclosed in a gray ellipse suggests the potential involvement of additional regulatory factors cooperating with NRCX in immune regulation

This study identifies a paired NLR system, NRCX/NARY, that co-regulates both growth and *P. capsici* resistance in *N. benthamiana* (Fig. 6). NLRs commonly function cooperatively within intricate signaling networks (Contreras et al. 2023). Canonical paired NLRs, typically genomically adjacent, include rice RGA5/RGA4 and Pik-1/Pik-2, which mediate resistance to *Magnaporthe oryzae* (Cesari et al. 2014; De la Concepcion et al. 2021; Zdrzalek et al. 2020), and Arabidopsis RRS1/RPS4, which confers immunity against *Pseudomonas syringae* (Narusaka et al. 2009). Similarly, the wheat NLR pair RXL/Pm5e confers powdery mildew resistance (Guo et al. 2025). Silencing experiments revealed distinct functional divergence: while knockout of one component of rice NLR pairs often induces lesion-mimicking or dwarfing phenotypes (Wang et al. 2019), *N. benthamiana* exhibits severe developmental defects exclusively upon NRCX silencing (Adachi et al. 2023).

The NRCX/NARY pair functions primarily as immune modulators. Canonical paired NLRs exhibit distinct roles, where sensor NLRs recognize effectors via integrated domains (e.g., HAM or WRKY), while executors activate immune responses, including cell death (De la Concepcion et al. 2021; Kanzaki et al. 2012; Sarris et al. 2015; Zdrzalek et al. 2020). However, neither NRCX nor NARY contains effector-recognition domains such as HAM or WRKY. Notably, mutations in the Walker B motif (hhhDD/E) and MHD motif of NARY failed to induce a hypersensitive response in *N. benthamiana* (Gao et al. 2011; Wang et al. 2015; Williams et al. 2011; Wu et al. 2019). Despite possessing a non-canonical Walker B (LIVLEDV) and MHD motif (LVG), NARY cannot trigger cell death, further supporting its role as a modulator rather than an executor. Nevertheless, NRCX/NARY retains key features of paired NLRs: (1) their "head-to-head" genomic arrangement, (2) the induction of a dwarf phenotype upon *NRCX* knockout—a hallmark of sensor NLR disruption, contrasting with the typical helper-NRC role, and (3) their interaction via CC domains, as observed in other paired NLR systems (Guo et al. 2025).

Beyond NRC2, NRC3, and NARY, additional factors influence the dwarfing and resistance phenotypes observed in *NRCX* knockouts. Adachi et al. reported that silencing *NRC2* and *NRC3* partially rescues the dwarf phenotype caused by *NRCX* silencing (Adachi et al. 2023). Our study further reveals that co-silencing *NARY* significantly enhances this phenotypic rescue. Parallel studies in Arabidopsis revealed analogous complexity: while knockout of *EDS1* fully rescues the dwarf phenotype of *snc1* mutants, simultaneous knockout of *SAG101-1* and *NRG1 A/B/C* only partially restores growth, indicating non-redundant roles for these genes in modulating immunity-associated developmental trade-offs (Wu et al. 2022).Similarly, in our study, simultaneous silencing of *NRC2, NRC3, NARY*, and *NRCX* failed to fully restore the wild-type phenotype in *N. benthamiana*, implying the involvement of additional factors or regulatory layers.

The immune signaling pathway regulated by NRCX/NARY warrants further investigation. In Solanaceae, lineage-specific expansions of NRC helper networks and associated sensor NLRs exemplify functional specialization (Goh et al. 2024; Wu et al. 2017). NRCX likely forms resistosomes as an NRC family member (Liu et al. 2024; Ma et al. 2024). However, NLRs may also form hetero-oligomeric complexes, as demonstrated by the paired immune receptors CHS3-CSA1 in plants (Yang et al. 2024) and the wheat NLR pair RXL/Pm5e, which preferentially forms hetero-complexes via CC-domain interactions (Guo et al. 2025). The Pikm-1 CC domain binds AvrPik for effector recognition (Kanzaki et al. 2012). In this study, NRCX self-interaction was diminished in the presence of NARY, suggesting that NRCX/NARY-mediated immunity may operate through CC-domain interactions. However, the absence of identified effectors or cell death induction leaves unresolved whether these NLRs function via hetero- or homo-complex formation.

Post-translational modifications may further regulate NLR activity. For instance, the Arabidopsis RRS1/RPS4 complex undergoes ubiquitination of RRS1’s WRKY domain by the E3 ligase RARE, destabilizing the complex and attenuating immune signaling (Chen et al. 2025). Phosphorylation has also been implicated in NLR signaling (Guo et al. 2020; Zhong et al. 2025). Whether NRCX/NARY activity is modulated by such mechanisms remains an open question.

## Conclusion

In conclusion, our work redefines the functional scope of paired NLRs by identifying NRCX/NARY as a non-canonical regulatory module. This NLR pair employs divergent motifs and CC domain-mediated interactions to fine-tune immunity without triggering cell death, distinct from both helper networks and classical effector-sensor pairs. These findings reveal a novel mechanism for calibrating immune responses through structural innovation, expanding the known functional diversity of NLR systems in balancing defense and growth.

## Meterials and methods

### Plant materials and growth conditions

Wild-type *N. benthamiana*, *NRCX* knockout lines, *NARY* knockout lines, and *NRCX/NARY* knockout lines were grown in a greenhouse under a 16-h photoperiod at 25℃ with 55–60% humidity. VIGS-treated *N. benthamiana* plants were grown in a controlled environment room at 22℃ with a 16-h light/8-h dark cycle and 55–60% relative humidity.

### *P. capsici* culture conditions and inoculation assays

The *P. capsici* strain LT263 was cultured and maintained at 25℃ in the dark on 10% (v/v) V8 agar plates. For inoculation assays, mycelium blocks were placed on the abaxial surface of leaves, with 0.1% Tween 20 added at the intersection of the mycelium and leaf tissue. The leaves were incubated in the dark at 25℃ for 24–36 h. Photos were taken under UV light, and the damaged area was quantified using ImageJ software.

### Plasmid construction

All plasmids and primers used for recombinant constructs in this study are listed in Table S1. NRCX and NARY were amplified from *N. benthamiana* genomic DNA (gDNA) and cloned into vectors such as pCambia1300-3xHA and pCambia1300-3xFlag. Primers used for cloning NRCX, NARY, NARY variants, and NRC3 variants are also provided in Table S1. Functional analyses of NARY and NRC3 were performed with untagged variants, while C-terminally tagged HA or FLAG variants produced consistent results in complementation assays.

### Virus-induced gene silencing (VIGS)

VIGS experiments were conducted in *N. benthamiana* as previously described (Ratcliff et al. 2001). Binary constructs pTRV2 were transformed into *Agrobacterium* strain GV3101. The constructs were incubated at 30℃ and 220 rpm for 24 h before infiltration. *Agrobacterium* cells were collected by centrifugation at 4,000 rpm for 4 min, washed and resuspended in infiltration buffer [10 mM MgCl_2_, 10 mM MES (pH 5.7) and 200 μM acetosyringone], The optical density (OD_600_) was adjusted to 0.5. Two-week-old *N. benthamiana* plants were infiltrated with a 1:1 suspension of Agrobacterium carrying TRV RNA1 and TRV RNA2.

### Transient gene expression and cell death assays

Binary expression plasmids were introduced into *Agrobacterium* strain GV3101 by electroporation. Four-week-old *N. benthamiana* plants were used for transient expression. *Agrobacterium* suspension was prepared as described in the VIGS protocol above.

### RT-qPCR analysis

Total RNA was extracted from plants using a RNA-simple Total RNA Kit (Tiangen Biotech Co., Ltd., Beijing, China). DNA contamination was removed using 4 × gDNA wiper (Vazyme Biotech Co., Ltd., Nanjing, China). For cDNA synthesis, 1 μg of RNA was reverse transcribed using HiScript II Q RT SuperMix for qPCR (Vazyme Biotech Co., Ltd., Nanjing, China). Real-time PCR was performed on an ABI Prism 7500 Fast Real-Time PCR System using the SYBR Premix Ex Taq kit (Takara Bio Inc., Shiga, Japan) following the manufacturer’s instructions. Gene expression levels were normalized to *NbEF1a*, a stably expressed reference gene in *N. benthamiana*. The primers used for RT-PCR are listed in Table S1. 

### Cultivation of *N. benthamiana* seedlings on 1/2 MS medium

Tobacco seeds (60) were sterilized by sequential treatment with 75% ethanol for 30 s, followed by three washes with sterile water. Seeds were then disinfected with 2.5% sodium hypochlorite for 8 min and washed six times with sterile water. The seeds were evenly distributed on 1/2 MS medium (0.8% agar, pH 5.7) and sealed. The medium was placed horizontally at 4℃ for 3 days to break dormancy, then transferred to the greenhouse to promote germination. Seedlings were photographed after 2–3 weeks.

### Protein extraction of *N. benthamiana* leaves

For western blot analysis, protein extraction was performed using buffer (50 mM HEPES, 150 mM KCL, 1 mM EDTA, and 0.1% Triton X-100; pH 7.5), supplemented with 1 mM DL-Dithiothreitol (DTT) and a protease inhibitor cocktail (Sigma-Aldrich, St. Louis, MO, USA). *N. benthamiana* leaves were frozen in liquid nitrogen, ground to a fine powder, and 1 mL of extraction buffer was added per 0.5 g of tissue. The mixture was vortexed, incubated at 4℃ for 30 min to allow full lysis, and then centrifuged at 13,000 rpm for 15 min at 4℃. To prepare samples for SDS-PAGE, 80 μL of 5 × sample loading buffer was added, mixed, and same protocol was followed for input samples, and the remaining supernatant was incubated with target beads.

### Western blot

Protein samples were separated by SDS-PAGE and transferred to a PVDF membrane using eBlot™ L1 (GenScript Corporation). Anti-HA (1:5,000; #M20013; Abmart Inc., Shanghai, China) and anti-FLAG (1:5,000; #M20018; Abmart) antibodies were used to detect proteins with the corresponding tags. Total protein levels were assessed by Ponceau staining.

### Split-luciferase assay

The coding sequences of the indicated genes were cloned into pCAMBIA1300-35S-HA-Nluc-RBS or pCAMBIA1300-35S-Cluc-RBS vectors and transferred into *A. tumefaciens* strain GV3101. Constructs were co-expressed in *N. benthamiana* plants (OD_600_ = 0.5). At 2 days post-infiltration (dpi), the leaves were treated with 1 mM luciferin (Biovision) and luciferase activity was measured using a microplate reader (BioTek, Beijing, China).

### Yeast two-hybrid system

pGBKT7-Bait or pGADT7-Prey constructs were transformed into the yeast strain AH109. Co-transformed yeast was plated on double dropout medium (SD-LW) and incubated at 28℃ for 3 days. Colonies that grew on SD-LW plates were considered positive for successful co-transformation. Subsequently, yeast colonies were diluted to OD_600_ = 0.1, followed by tenfold and 100-fold dilutions. 10 μL of each dilution was spotted on SD-LW and triple dropout (SD-LWH) plates, and images were taken after incubation at 28℃ for 3 days.

### Phylogenetic analysis

Multiple sequence alignments of full-length amino acid sequences were performed using MUSCLE. Phylogenetic analysis of NB-ARC sequences was conducted using the FastTree program or MEGA X program, with the maximum likelihood method, 100 bootstrap samples, and the following parameters: Poisson model, uniform rates, and complete deletion.

### Accession number

The accession numbers for the sequences supporting this study are as follows: NbD037153.1, NbD002668.1, NbD002669.1, NbD026701.1, NbD026704.1, NbD022578.1 (NARY), and NbD022579.1 (NRCX), all from *Nicotiana benthamiana* in the Oxford University Research Archive (ORA).

## Supplementary Information

Additional file 1: Figure S1**. **Virus-induced silencing of *NRCX* impairs growth in *N. benthamiana*. **A** Phenotypes of 6-week-old *NbD037153.1*-silenced and *NRCX*-silenced *N. benthamiana* plants. TRV constructs were agroinfiltrated at the two-leaf stage, with TRV-GUS as a control. Bar = 2 cm. **B** Silencing efficiency of *NbD037153.1* and *NRCX* quantified by RT-qPCR (means ± SD; *n* = 3 technology replicates). **C** Phylogenetic tree of *NRCX* homologs in *N. benthamiana*. Bootstrap values (> 70%) from 1,000 replicates are shown at branch nodes. **D** Flanking sequence of the potential CRISPR target. The *nrcx* mutant contains a 7-bp deletion in the *NRCX* coding sequence, with no off-target mutations detected at predicted sites.

Additional file 2: Figure S2. Summary of predicted NLRs. The phylogeny of the 29 species is based on data from the Taxonomy Database and previous studies (Ngou et al. 2022). The numbers of NLRs, paired NLRs and NRC helper NLRs in each species are shown in boxplots alongside the species names. NLRs were identified from plant proteomes using NLRtracker (Kourelis et al. 2021). NLRtracker hits were annotated as NB-ARC domain-containing proteins that were considered NLRs. Paired NLRs were defined as NLRs in a “head-to-head” arrangement, separated by no more than two non-NLR genes. NRC helper NLRs were identified through phylogenetic analysis (Wu et al. 2017). NLRs that clustered with functionally validated NRC helpers (NRC2, NRC3, and NRC4) were classified as NRC helper NLRs.

Additional file 3: Figure S3. Silencing of paired NLRs in *N. benthamiana*. **A** Genomic organization of six paired NLRs in *N. benthamiana.* **B** Phenotypes of *N. benthamiana* plants following VIGS of six NLR partners. TRV constructs were agroinfiltrated into two-week-old plants, and phenotypes were recorded 4 weeks post-infiltration. TRV:*GUS* (β-glucuronidase) served as a control. Bar = 2 cm. **C** Silencing efficiency of *NRCX* quantified by RT-qPCR. TRV:*GUS*-infiltrated plants were used as controls.

Additional file 4: Figure S4. Co-silencing *NARY* partially rescues the dwarf phenotype of *NRCX*-silenced plants.** A** Genomic loci of *NRCX* and *NARY*, separated by 18,795 bp. Exons (green, yellow, pink for CC, NB-ARC, and LRR domains, respectively) and introns are annotated. Translation start sites are marked with inverted triangles. **B** TRV vector designs for *NRCX* (deep red) and *NARY* (deep blue) silencing. Target regions (~ 300 bp) are highlighted. **C** Silencing efficiency of *NRCX* and *NARY* (mean ± SD; *n* = 3; **, *P* < 0.01, Student’s *t*-test). **D** Phenotypes of *N. benthamiana* plants silenced for *NRCX* (TRV-*X*), *NARY* (TRV-*Y*), or both (TRV-*X/Y*). TRV-*GUS* was the control. Bars = 2 cm. **E** Leaf diameter measurements (mean ± SD; *n* = 16; lowercase letters indicate significant differences by one-way ANOVA, **, *P* < 0.01). **F** *PR1* expression in indicated leaves (mean ± SD; *n* = 3; **, *P* < 0.01, Student’s *t*-test).

Additional file 5: Figure S5. Mutation sites in *nrcx/nary* (*xy)* and *nary (y)* knockout lines. **A,B** Genomic structures of *nary* (blue) and *nrcx* (gray) in *N. benthamiana*. gRNA target sites (bright yellow) and sequencing-confirmed mutations (red) are shown for wild-type, *xy* (double knockout), and *y* (*nary* single knockout) lines.

Additional file 6: Figure S6. Phenotypes of plants with simultaneous silencing of NRC2/3, NARY and NRCX. **A** Growth phenotypes of wild-type *N. benthamiana* subjected to VIGS targeting *NRC2/NRC3* (TRV-*2/3*), *NRC2/NRC3/NRCX* (TRV-*2/3/X*), or *NRC2/NRC3/NRCX/NARY* (TRV-*2/3/X/Y*). Plants were agroinfiltrated with TRV constructs at the two-leaf stage (2-week-old) and imaged 9 weeks post-treatment. Bars = 1 cm. **B** Phenotypes of detached leaves from silenced plants. Leaves (third to fourth true leaves from the apex) were excised and arranged for comparison. Bar = 1 cm. **C** Quantification of leaf diameter. Data represent mean ± SD (*n* = 18); lowercase letters denote significant differences between groups (one-way ANOVA, *P* < 0.05). **D** RT-qPCR analysis of target gene silencing efficiency. Expression levels of *NRC2*, *NRC3*, *NRCX*, and *NARY* were normalized to the internal control *EF1a*. TRV-*GUS*-infiltrated plants served as negative controls. **E** Phenotypes of *NRCX/NARY*-silenced wild-type and *nrc2/3/4* triple knockout mutants. Plants were treated and imaged as in (A). Bars = 1 cm.

Additional file 7: Figure S7. Phylogenetic analysis of NARY homologs. A maximum-likelihood phylogenetic tree (RAxML v8.2.12, JTT model) of NB-ARC domains from 2189 NLRs across *Solanum lycopersicum* (Solyc-), *Nicotiana benthamiana* (NbD-), coffee (*Coffea canephora*, Cc-), kiwifruit (*Actinidia deliciosa*, DTZ-), sugar beet (*Beta vulgaris*, EL-), *Arabidopsis thaliana* (AT-), and rice (*Oryza sativa*, Os-). The NARY-clade (blue) and NRCX-clade (orange) are highlighted. Domain architectures and conserved motifs (identified by MEME) of the NARY-clade are expanded at bottom right.

Additional file 8: Table S1. List of primers in this article.

## Data Availability

All data and materials are available in the paper and online supplemental files.

## Abbreviations

*NARY*: *NRCX* Adjacent resistance gene Y
ETI: Effector-triggered immunity
HR: Hypersensitive response
CC: Coiled-coil
TIR: Toll/interleukin-1 receptor domain
NRC: NLR-required for cell death
NB-ARC: Nucleotide-binding (NB)-ARC (APAF1, R gene products, and CED-4) domain
LRR: Leucine-rich repeat
ID: Integrated decoy
Sensor NLR: An NLR (nucleotide-binding leucine-rich repeat receptor) responsible for identifying effectors
Helper NLR: A downstream NLR involved in sensor NLR-mediated immunity
VIGS: Virus-induced gene silencing
SD Media: Synthetic dropout media
