# Supplementary figures and images for: A CNL protein forms an NLR pair with NRCX to modulate plant immunity
Source: Stress Biol. 2025 Sep 3;5(1):55. doi: 10.1007/s44154-025-00245-6 (PMC12405136; doi:10.1007/s44154-025-00245-6)

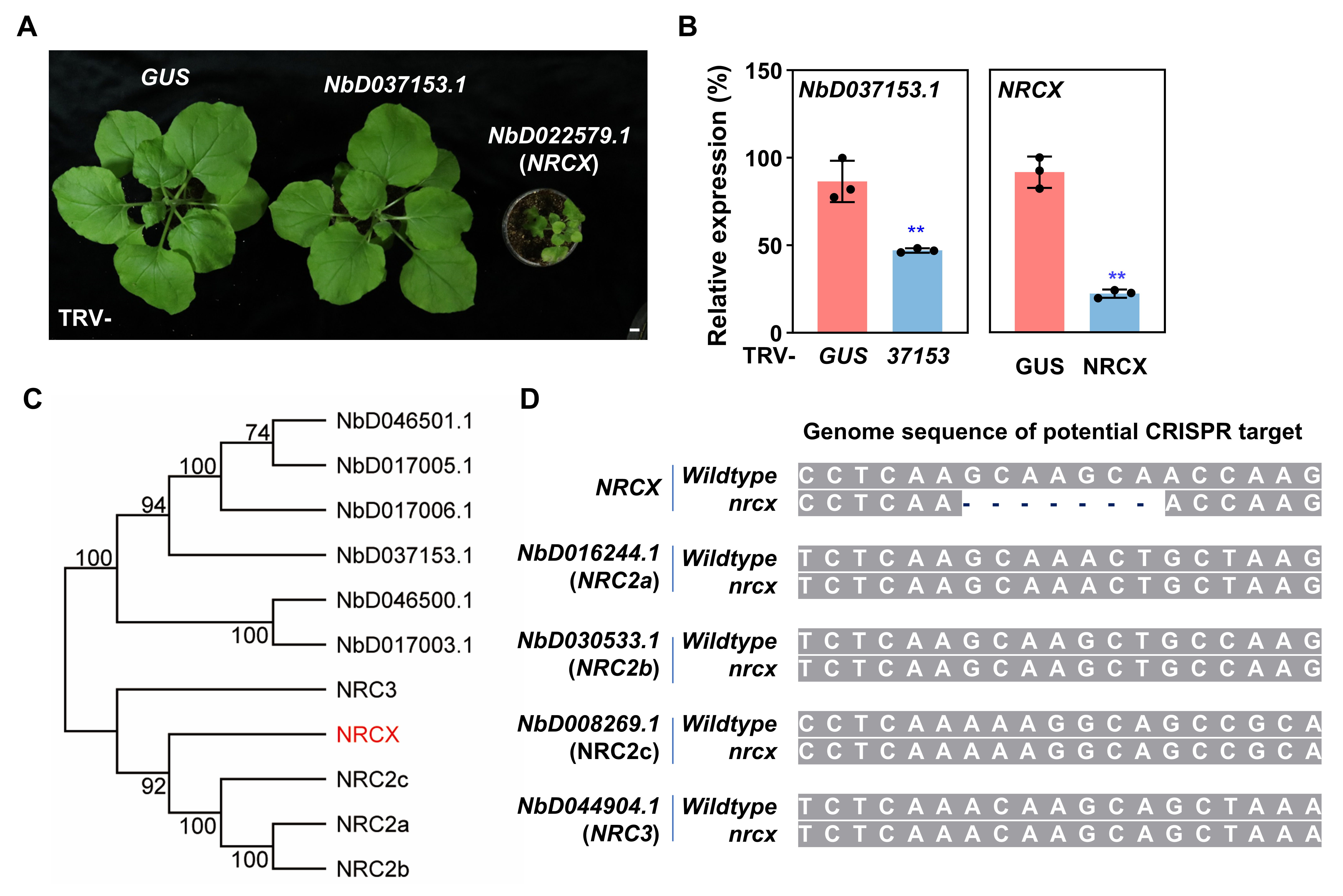

Supplement: Supplementary file 1 — Additional file 1: Figure S1. Virus-induced silencing of NRCX impairs growth in N. benthamiana. A Phenotypes of 6-week-old NbD037153.1-silenced and NRCX-silenced N. benthamiana plants. TRV constructs were agroinfiltrated at the two-leaf stage, with TRV-GUS as a control. Bar = 2 cm. B Silencing efficiency of NbD037153.1 and NRCX quantified by RT-qPCR (means ± SD; n = 3 technology replicates). C Phylogenetic tree of NRCX homologs in N. benthamiana. Bootstrap values (> 70%) from 1,000 replicates are shown at branch nodes. D Flanking sequence of the potential CRISPR target. The nrcx mutant contains a 7-bp deletion in the NRCX coding sequence, with no off-target mutations detected at predicted sites. [file 44154_2025_245_MOESM1_ESM.tif]

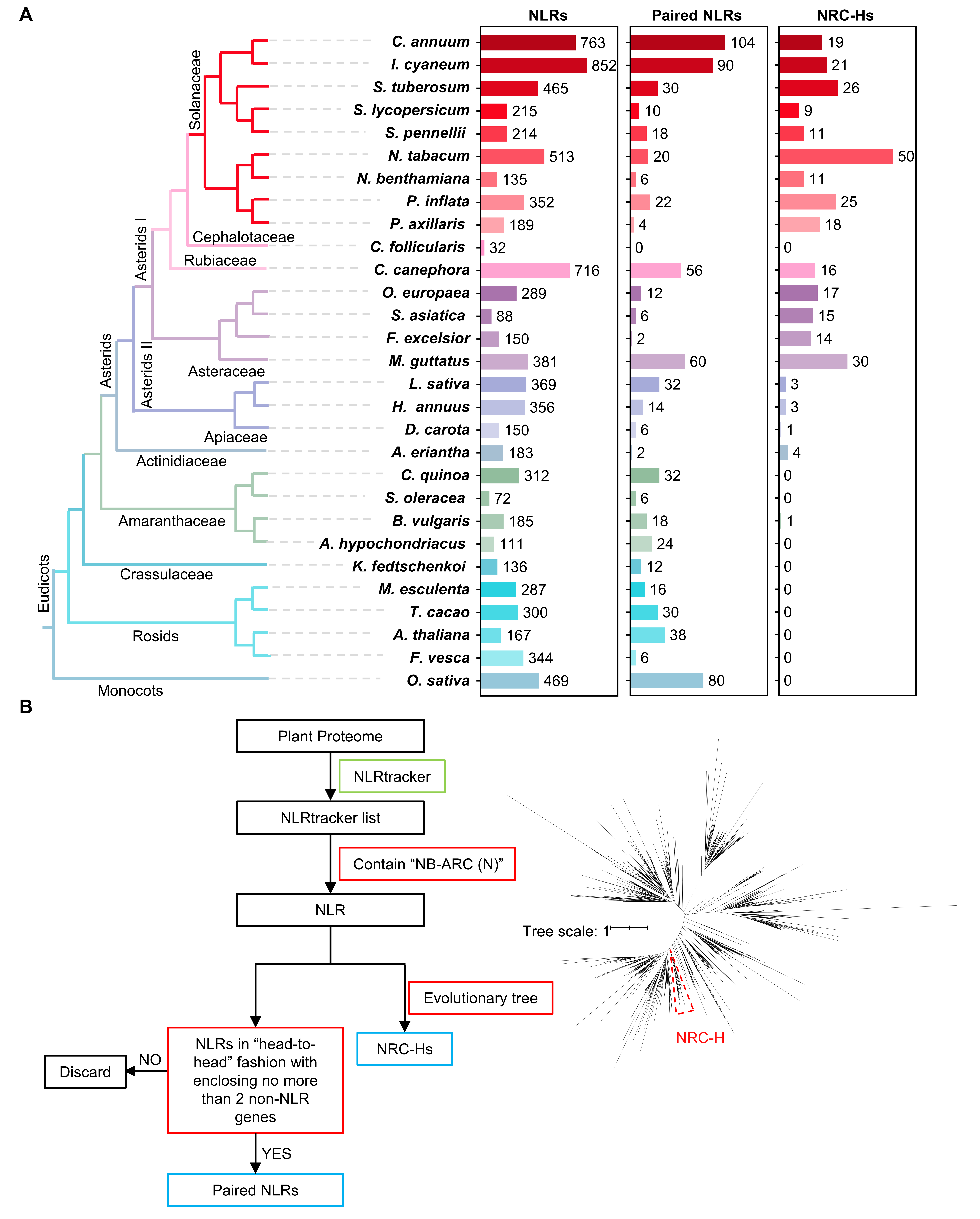

Supplement: Supplementary file 2 — Additional file 2: Figure S2. Summary of predicted NLRs. The phylogeny of the 29 species is based on data from the Taxonomy Database and previous studies (Ngou et al. 2022). The numbers of NLRs, paired NLRs and NRC helper NLRs in each species are shown in boxplots alongside the species names. NLRs were identified from plant proteomes using NLRtracker (Kourelis et al. 2021). NLRtracker hits were annotated as NB-ARC domain-containing proteins that were considered NLRs. Paired NLRs were defined as NLRs in a “head-to-head” arrangement, separated by no more than two non-NLR genes. NRC helper NLRs were identified through phylogenetic analysis (Wu et al. 2017). NLRs that clustered with functionally validated NRC helpers (NRC2, NRC3, and NRC4) were classified as NRC helper NLRs. [file 44154_2025_245_MOESM2_ESM.tif]

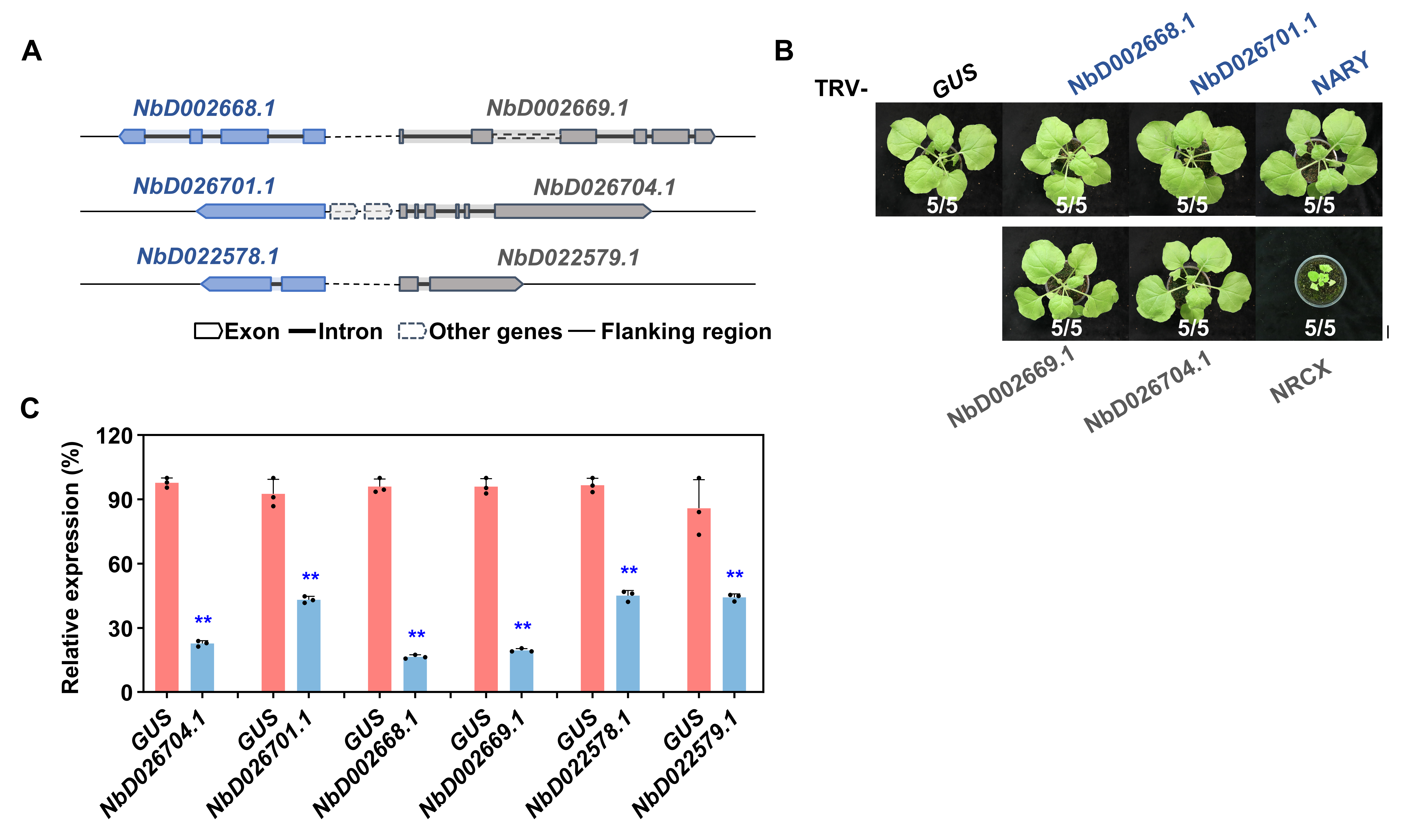

Supplement: Supplementary file 3 — Additional file 3: Figure S3. Silencing of paired NLRs in N. benthamiana. A Genomic organization of six paired NLRs in N. benthamiana. B Phenotypes of N. benthamiana plants following VIGS of six NLR partners. TRV constructs were agroinfiltrated into two-week-old plants, and phenotypes were recorded 4 weeks post-infiltration. TRV:GUS (β-glucuronidase) served as a control. Bar = 2 cm. C Silencing efficiency of NRCX quantified by RT-qPCR. TRV:GUS-infiltrated plants were used as controls. [file 44154_2025_245_MOESM3_ESM.tif]

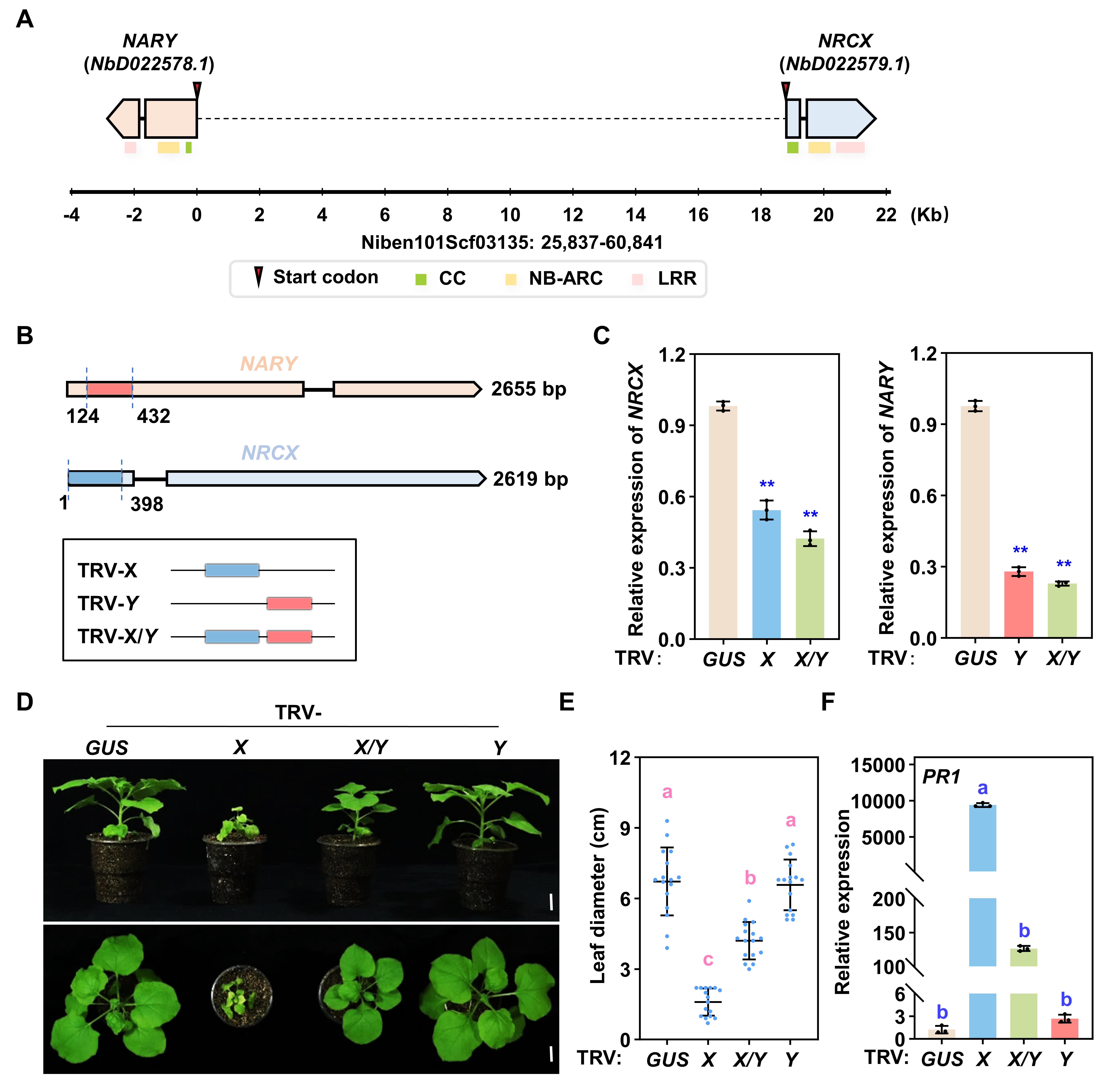

Supplement: Supplementary file 4 — Additional file 4: Figure S4. Co-silencing NARY partially rescues the dwarf phenotype of NRCX-silenced plants. A Genomic loci of NRCX and NARY, separated by 18,795 bp. Exons (green, yellow, pink for CC, NB-ARC, and LRR domains, respectively) and introns are annotated. Translation start sites are marked with inverted triangles. B TRV vector designs for NRCX (deep red) and NARY (deep blue) silencing. Target regions (~ 300 bp) are highlighted. C Silencing efficiency of NRCX and NARY (mean ± SD; n = 3; **, P < 0.01, Student’s t-test). D Phenotypes of N. benthamiana plants silenced for NRCX (TRV-X), NARY (TRV-Y), or both (TRV-X/Y). TRV-GUS was the control. Bars = 2 cm. E Leaf diameter measurements (mean ± SD; n = 16; lowercase letters indicate significant differences by one-way ANOVA, **, P < 0.01). F PR1 expression in indicated leaves (mean ± SD; n = 3; **, P < 0.01, Student’s t-test). [file 44154_2025_245_MOESM4_ESM.tif]

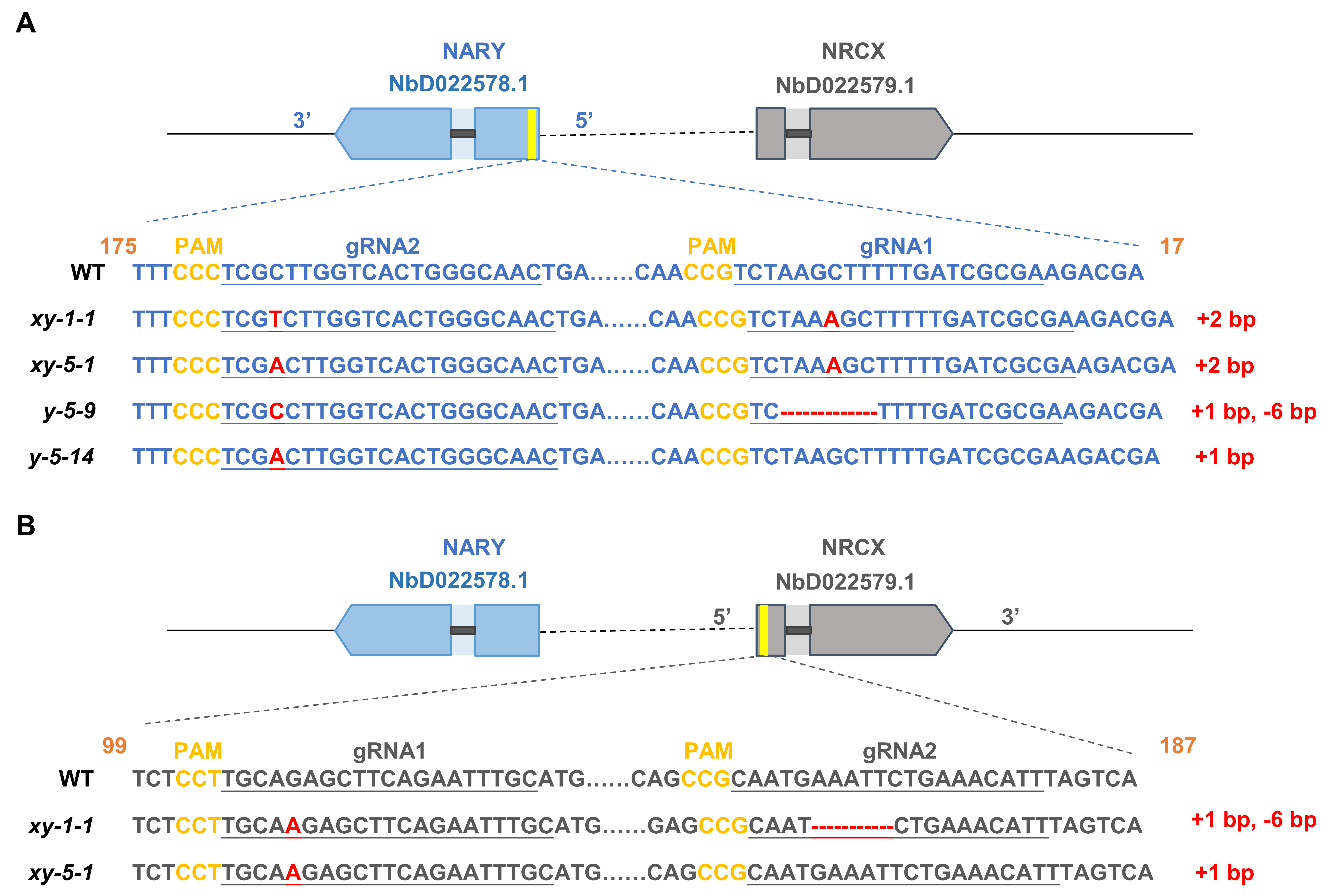

Supplement: Supplementary file 5 — Additional file 5: Figure S5. Mutation sites in nrcx/nary (xy) and nary (y) knockout lines. A,B Genomic structures of nary (blue) and nrcx (gray) in N. benthamiana. gRNA target sites (bright yellow) and sequencing-confirmed mutations (red) are shown for wild-type, xy (double knockout), and y (nary single knockout) lines. [file 44154_2025_245_MOESM5_ESM.tif]

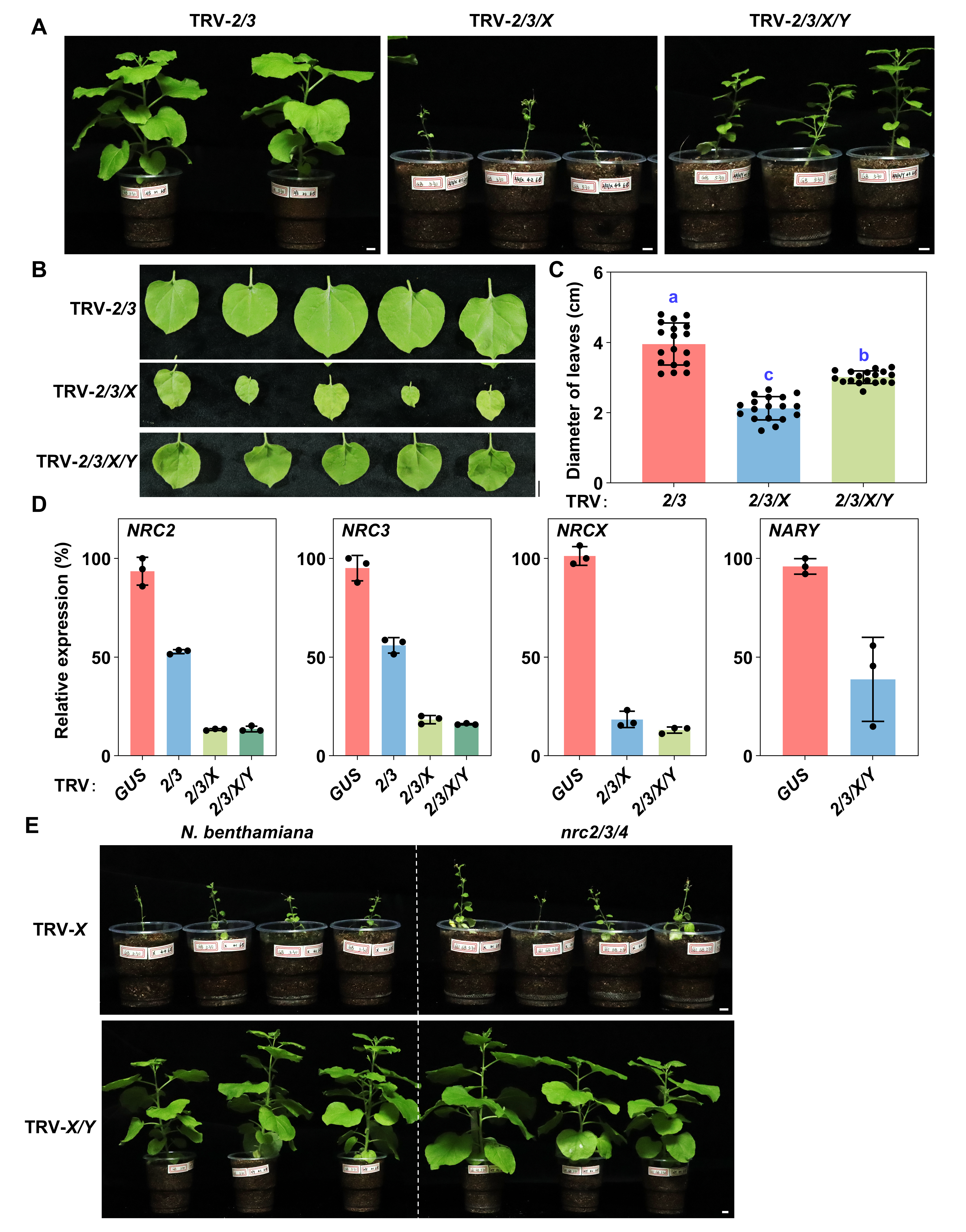

Supplement: Supplementary file 6 — Additional file 6: Figure S6. Phenotypes of plants with simultaneous silencing of NRC2/3, NARY and NRCX. A Growth phenotypes of wild-type N. benthamiana subjected to VIGS targeting NRC2/NRC3 (TRV-2/3), NRC2/NRC3/NRCX (TRV-2/3/X), or NRC2/NRC3/NRCX/NARY (TRV-2/3/X/Y). Plants were agroinfiltrated with TRV constructs at the two-leaf stage (2-week-old) and imaged 9 weeks post-treatment. Bars = 1 cm. B Phenotypes of detached leaves from silenced plants. Leaves (third to fourth true leaves from the apex) were excised and arranged for comparison. Bar = 1 cm. C Quantification of leaf diameter. Data represent mean ± SD (n = 18); lowercase letters denote significant differences between groups (one-way ANOVA, P < 0.05). D RT-qPCR analysis of target gene silencing efficiency. Expression levels of NRC2, NRC3, NRCX, and NARY were normalized to the internal control EF1a. TRV-GUS-infiltrated plants served as negative controls. E Phenotypes of NRCX/NARY-silenced wild-type and nrc2/3/4 triple knockout mutants. Plants were treated and imaged as in (A). Bars = 1 cm. [file 44154_2025_245_MOESM6_ESM.tif]

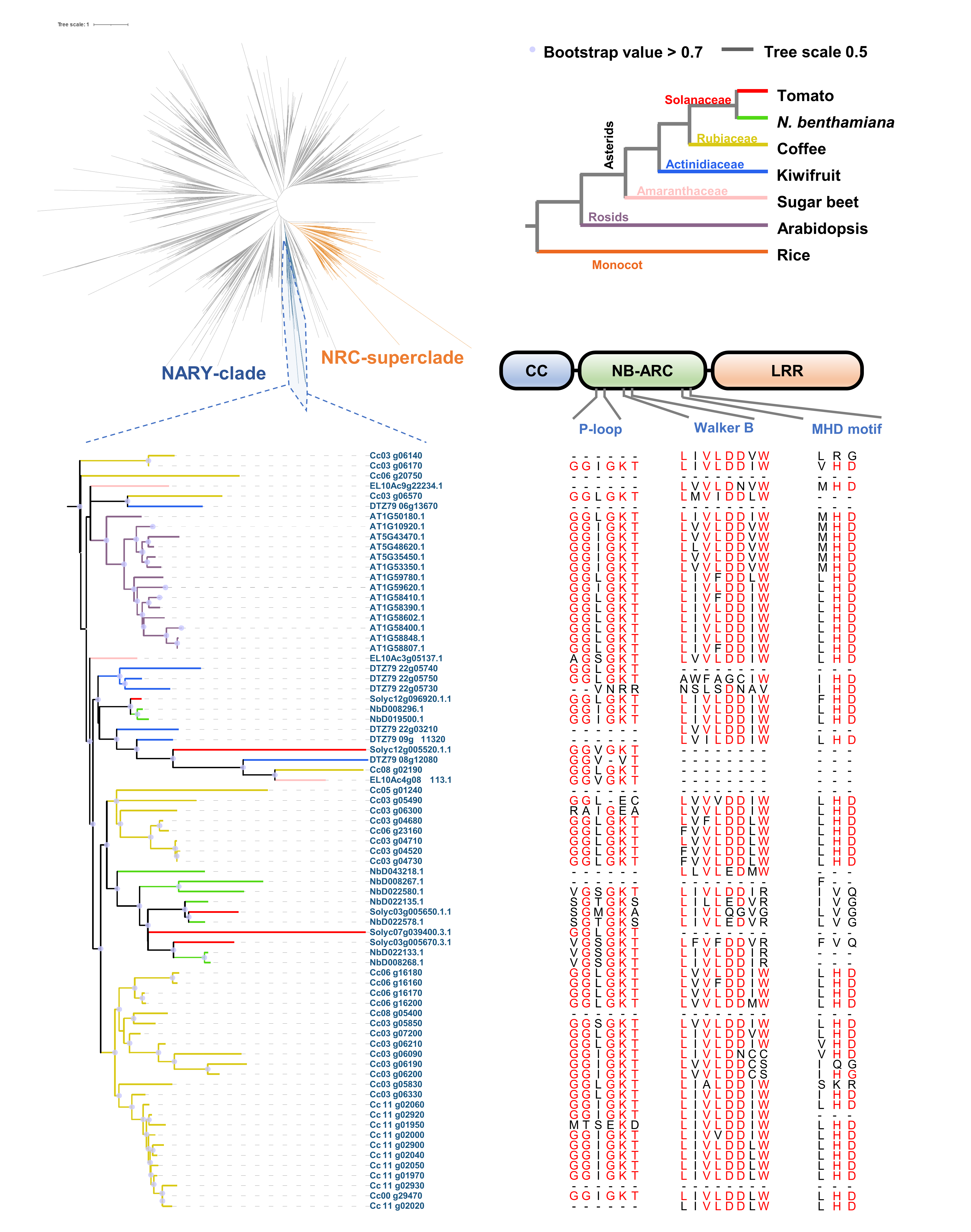

Supplement: Supplementary file 7 — Additional file 7: Figure S7. Phylogenetic analysis of NARY homologs. A maximum-likelihood phylogenetic tree (RAxML v8.2.12, JTT model) of NB-ARC domains from 2189 NLRs across Solanum lycopersicum (Solyc-), Nicotiana benthamiana (NbD-), coffee (Coffea canephora, Cc-), kiwifruit (Actinidia deliciosa, DTZ-), sugar beet (Beta vulgaris, EL-), Arabidopsis thaliana (AT-), and rice (Oryza sativa, Os-). The NARY-clade (blue) and NRCX-clade (orange) are highlighted. Domain architectures and conserved motifs (identified by MEME) of the NARY-clade are expanded at bottom right. [file 44154_2025_245_MOESM7_ESM.tif]
